# Emphasizing the O in OPAT: A Pathway for Clinic-Initiated Outpatient Parenteral Antimicrobial Therapy (CI-OPAT) at an Academic Center

**DOI:** 10.1093/ofid/ofag105

**Published:** 2026-03-03

**Authors:** Molly McDonough, Michael Yarrington, Jason Funaro, Jenny Shroba, Kristen Dicks

**Affiliations:** Department of Medicine, Division of Infectious Diseases, Duke University Medical Center, Durham, North Carolina, USA; Department of Medicine, Division of Infectious Diseases, Duke University Medical Center, Durham, North Carolina, USA; Department of Medicine, Division of Infectious Diseases, Duke Center for Antimicrobial Stewardship and Infection Prevention, Durham, North Carolina, USA; Department of Pharmacy, Duke University Hospital, Durham, North Carolina, USA; Department of Pharmacy, Duke University Hospital, Durham, North Carolina, USA; Department of Medicine, Division of Infectious Diseases, Duke University Medical Center, Durham, North Carolina, USA

**Keywords:** clinic-initiated OPAT, OPAT, outpatient parenteral antimicrobial therapy

## Abstract

Our infectious diseases (ID) clinic began a clinic-initiated outpatient parenteral antimicrobial therapy (CI-OPAT) program to avoid unnecessary emergency department visits or inpatient admissions. In this single-center retrospective case series, we describe the treatment of 59 outpatients with CI-OPAT with low rates of complications. These findings suggest that CI-OPAT programs can be safe and effective.

## Teaser

Our infectious diseases clinic implemented a program for clinic-initiated OPAT (CI-OPAT) to avoid unnecessary emergency department visits and inpatient admissions. This single-center retrospective case series describes the treatment of 59 outpatients with CI-OPAT with low rates of complications suggesting that CI-OPAT programs can be safe and effective for appropriately selected patients.

Outpatient parenteral antimicrobial therapy (OPAT) is the administration of parenteral antimicrobials in care settings outside the hospital. In the United States, OPAT is typically administered at the patient's home, in a skilled nursing facility, or at an ambulatory infusion center. Benefits of OPAT include earlier hospital discharges with associated cost savings, patient satisfaction, and fewer hospital-acquired complications. Adverse events can also occur, such as drug reactions, line complications, and treatment failure, resulting in readmissions, morbidity, and deaths [1].

Existing OPAT studies primarily describe outcomes in patients discharged from the hospital on OPAT to complete therapy [2–5]. Less is known about the safety of OPAT initiated in clinic or ambulatory care settings. Initiation of OPAT in outpatient settings provides an opportunity to avoid unnecessary emergency department visits and inpatient admissions.

In 2022, our clinic began a formal program for clinic-initiated OPAT (CI-OPAT) through an interprofessional collaboration of providers, OPAT pharmacists, and vascular access nurses in our infectious diseases (ID) clinic. The objective of the current study was to describe this program and review its safety outcomes.

## METHODS

### Context

Our OPAT team comprises 2 pharmacists, a nurse coordinator, and a part-time medical director. This team manages approximately 1375 patients per year who are discharged from the hospital on OPAT. The average weekly census in the program is 170 patients. Our ID practice consists of 35 providers practicing at 4 locations in Durham, North Carolina. The primary ID clinic is located within the academic medical center. A unique feature of our clinic is 3 full-time nurses who are trained in vascular access and can place or troubleshoot peripherally inserted central catheters (PICCs) and midline catheters when needed for OPAT patients but who otherwise fill routine ID clinic roles. The lines are placed in a designated procedure room in the ID clinic. The program required purchase of an ultrasonography machine for vascular access placement and a vascular positioning system device that uses endovascular electrocardiography to confirm device placement.

**Figure 1.**
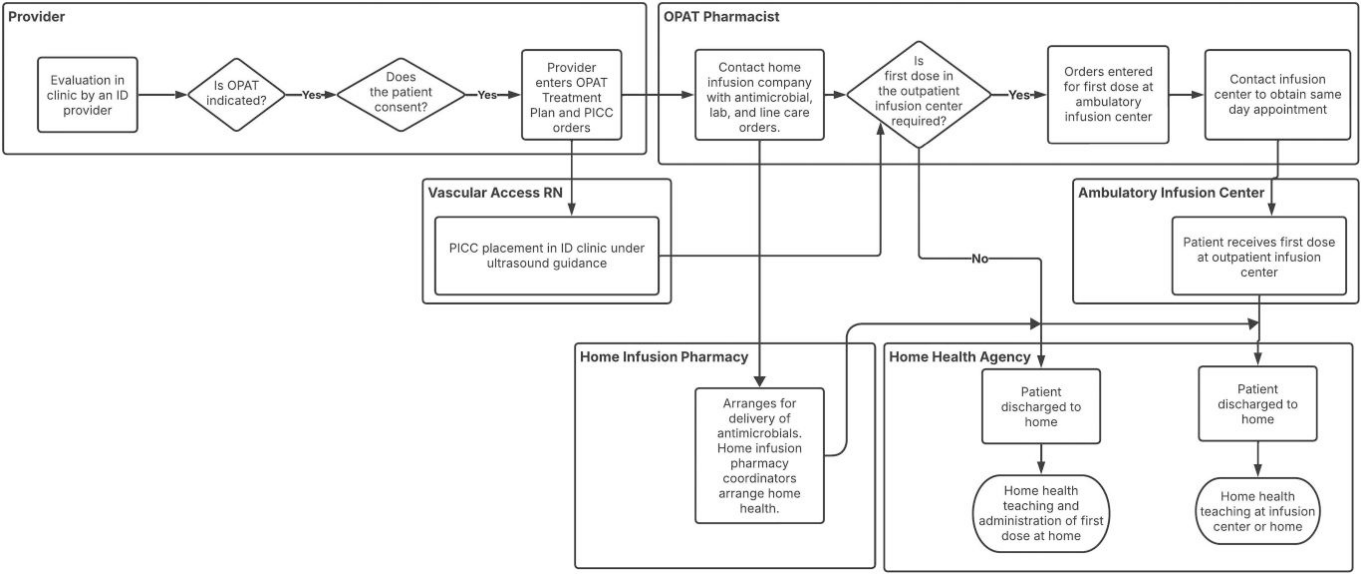
Process map for clinic initiation of outpatient parenteral antimicrobial therapy (OPAT). Abbreviations: ID, infectious diseases; PICC, peripherally inserted central catheter; US, ultrasonography.

The CI-OPAT process in our clinic is initiated by providers with assistance from pharmacists and with line placement by these vascular access nurses where applicable (Figure 1). Patients are required to be clinically stable and able to provide informed consent (or have a surrogate decision maker who is designated to provide informed consent) for line placement and OPAT treatment. Patients who have not previously received the treatment medication are required to receive an observed first dose in our hospital's outpatient infusion center or with a home health nurse equipped with an anaphylaxis kit.

### Study Design and Analysis

In this single-center retrospective case series, we reviewed all adult patients who received CI-OPAT in our general ID OPAT program from 2 July 2022 through 30 June 2023. This study was approved by the Duke University Institutional Review Board. Patient consent was not applicable to this report.

Cases were reviewed to evaluate outcomes, including hospital admission while receiving OPAT, serious vascular access complications, and adverse drug events (ADEs). OPAT-related admissions were defined as admissions for therapeutic failure after source control requiring a change in antimicrobials or vascular access complications or ADEs requiring hospital admission. Vascular access complications were defined as central line–associated bloodstream infection or line-associated thrombus requiring anticoagulation. ADEs were defined as clinical symptoms or laboratory abnormalities that resulted in a change in therapy.

Patients were identified using OPAT care episodes in our healthcare system's electronic health record, Epic [6, 7]. Patients were excluded if they received OPAT that was not monitored by our OPAT program (patients receiving hemodialysis, solid organ transplant recipients, patients with hematologic cancers, or patients with ventricular assist devices). Demographic data was extracted from electronic health records using the Duke Enterprise Data Unified Content Explorer (DEDUCE) [8]. Data regarding prescribed antimicrobials and indications for therapy were extracted from provider documentation. *International Classification of Diseases, Tenth Revision* codes documented before the care episode were used to calculate the age-adjusted Charlson comorbidity index [9]. Data regarding PICC placement in the ID clinic, observed first infusions, OPAT complications, and admissions were extracted from patient records by manual review. Descriptive analysis was done using Microsoft Excel software, version 1808 (Microsoft).

## RESULTS

Our program provided CI-OPAT to 59 unique patients in the 1-year period of our study (Table 1). Most patients received parenteral antimicrobials at home with home healthcare, though 1 patient completed OPAT at a skilled nursing facility. The majority of CI-OPAT patients (n = 49 [83%]) had PICCs placed in the ID clinic. The vascular access nurses in our ID clinic can also place midline catheters, although patients in this study had PICCs placed due to the planned duration of treatment. Patients were most often treated for bone and joint infections (n = 34 [58%]), followed by pulmonary (n = 8 [14%]) and central nervous system (n = 7 [12%]) infections, which included neurosyphilis (n = 5) and cerebral abscess (n = 2). The most commonly prescribed antibiotics were β-lactams (n = 44 [75%]) and vancomycin (n = 13 [22%]), with 19 patients (32%) receiving combination therapy. Observed first doses occurred at the ambulatory infusion center for 20 patients (34%), in the home with a home health nurse equipped with an anaphylaxis kit for 9 patients (15%), and at a skilled nursing facility for 1 patient (2%). Twenty-nine patients (49%) did not receive a medically supervised first dose, typically due to previous exposure to the planned medication.

**Table 1.** Demographic and Clinical Patient Characteristics and Outcomes in Clinic-Initiated Outpatient Parenteral Antimicrobial Therapy

Characteristic or Outcome	Patients, No. (%)^a^ (N = 59)
Age, median (IQR), y	63 (50–73)
Age-adjusted CCI, median (IQR)	3 (2–5)
OPAT duration, median (IQR), d	42 (24–50)
Type of infection	
Bone/joint	34 (58)
Pulmonary	8 (14)
Central nervous system	7 (12)
Genitourinary	5 (8)
Skin/soft tissue	4 (7)
Other	1 (2)
Antibiotic	
β-lactam	44 (75)
Vancomycin	13 (22)
Aminoglycoside	7 (12)
Combination therapy	19 (32)
Catheter type	
PICC	57 (97)
Tunneled catheter	2 (3)
Port	0 (0)
PICC placement site	
ID clinic	49 (83)
Placed at time of outpatient surgery	3 (5)
Outpatient placement by hospital-based vascular access team	3 (5)
Tunneled line placed by interventional radiology	2 (3)
Line placed before ID clinic appointment	2 (3)
Administration of medically supervised first dose	
No	29 (49)
At outpatient infusion center	20 (34)
At home with a home health nurse	9 (15)
At a skilled nursing facility	1 (2)
Outcomes	
Unplanned admission while on OPAT	
Yes	5 (8)
No	54 (92)
Line complication	
Catheter-associated deep vein thrombosis	0 (0)
Central line–associated bloodstream infection	0 (0)
ADEs	
Gastrointestinal	3 (5)
Tinnitus	2 (3)
Thrombocytopenia	1 (2)
Neutropenia	1 (2)
Elevated liver enzymes	2 (4)
Myalgias	1 (2)
Total	10 (14)^b^

Abbreviations: ADEs, adverse drug events; CCI, Charlson comorbidity index; ID, infectious disease; IQR, interquartile range; OPAT, outpatient parenteral antimicrobial therapy; PICC, peripherally inserted central catheter.

^a^Data represent no. (%) of patients unless otherwise specified.

^b^There were a total of 10 ADEs in 8 patients.

There were no serious vascular access complications in this cohort (Table 1). Eight patients (14%) experienced a total of 10 ADEs requiring a change in therapy during their OPAT course (Table 2). A total of 5 patients (8%) required hospital admission during OPAT. These admissions were for additional source control measures or reasons unrelated to OPAT and did not require a change in antimicrobials (Table 3).

**Table 2.** Patients Who Experienced Adverse Drug Events While Receiving Clinic-Initiated Outpatient Parenteral Antimicrobial Therapy

Patient	Age, y	OPAT Indication	Initial Antimicrobial Regimen	ADEs	Updated Treatment Plan
1	69	Pulmonary NTM	Amikacin, tigecycline, imipenem-cilastatin	Nausea	Amikacin, imipenem-cilastatin, linezolid
2	73	Pulmonary NTM	Amikacin, tigecycline, imipenem-cilastatin	Nausea, weight loss	Treatment discontinued
3	76	Pulmonary NTM	Amikacin, imipenem-cilastatin, omadacycline	Tinnitus	Imipenem-cilastatin, omadacycline, clofazamine
4	19	Osteomyelitis	Ceftriaxone, metronidazole	Elevated liver enzymes	Amoxicillin-clavulanate
5	74	Osteomyelitis	Vancomycin, levofloxacin	Thrombocytopenia	Daptomycin, Levofloxacin
6	29	Septic arthritis	Ceftaroline, fluconazole	Neutropenia, elevated liver enzymes	Treatment discontinued
7	57	Septic arthritis	Daptomycin	Myalgias	Vancomycin
8	61	Prosthetic joint infection	Ceftriaxone	Diarrhea	Doxycycline

Abbreviations: ADEs, adverse drug events; NTM, nontuberculous mycobacteria; OPAT, outpatient parenteral antimicrobial therapy.

**Table 3.** Patients Admitted to the Hospital While Receiving Clinic-Initiated Outpatient Parenteral Antimicrobial Therapy

Patient	Age, y	OPAT Indication	Antimicrobial Regimen	Reason for Readmission	Change in Antimicrobials
1	35	Prosthetic joint infection	Vancomycin	Persistent infection	No
2	74	Thigh abscess	Amikacin, azithromycin, linezolid	Persistent infection	No
3	69	Prosthetic joint infection	Vancomycin	Nausea and vomiting	No
4	29	Septic arthritis	Ceftaroline	Acute kidney injury	No
5	71	Prosthetic joint infection	Ceftriaxone	Fever, cause undetermined	No

Abbreviation: OPAT, outpatient parenteral antimicrobial therapy.

## DISCUSSION

Our study demonstrates the feasibility and safety of CI-OPAT at an academic medical center. CI-OPAT involves close interprofessional collaboration between ID providers, OPAT pharmacists, home health agencies, home infusion pharmacies, and ambulatory infusion centers. A critical component of our program has been the implementation of trained vascular access nurses as part of our clinic staff. These nurses have the specialization required to place, replace, and troubleshoot midline catheters and PICCs, thus facilitating CI-OPAT initiation. This study adds to existing literature demonstrating the importance of collaborative interprofessional OPAT teams [10, 11]. To our knowledge, this is the first study demonstrating the safety of PICC placement by nurses with vascular access training embedded in an ID clinic.

The concern about starting a new antibiotic as an outpatient may be a barrier for some patients and providers to starting OPAT in the outpatient setting. OPAT guidelines from the Infectious Diseases Society of America advise that a first dose of a new antimicrobial may be administered with the supervision of a healthcare provider who can respond to an anaphylactic reaction [1]. One case series described immediate adverse reactions in 6 of 93 outpatients who received a supervised first-dose infusion, with no immunoglobulin E–mediated reactions. All 6 patients who had a reaction were able to complete their first dose and receive the planned course of the prescribed antimicrobial [12].

This case series demonstrates favorable safety outcomes for CI-OPAT. Only 5 of 59 CI-OPAT patients (8%) required admission during therapy (Table 3). This is lower than published 30-day readmission rates for patients discharged from the hospital on OPAT, which range from 18% to 26% [2, 4, 10, 13–18]. A prior study from our own institution demonstrated a 30-day unplanned readmission rate of 20% [19]. The current study demonstrates the relative safety of CI-OPAT in appropriately selected patients. There are likely several reasons for lower rates of complications among patients receiving CI-OPAT compared with conventional OPAT initiated in an inpatient setting. First, patients treated with CI-OPAT have more indolent and chronic infections without associated systemic symptoms, and this lower acuity permits the time and logistics required for OPAT initiation outside a hospital setting. Second, patients eligible for CI-OPAT are able to present to the clinic for expedited outpatient evaluation, line placement, and therapy initiation, which may reflect fewer medical or social barriers to receiving complex outpatient care. Third, our CI-OPAT program does not currently provide outpatient antimicrobial starts for patients who have received solid organ transplants or who have hematologic cancer or ventricular assist devices as these patients are managed by separate programs within our institution. The degree of influence of these clinical and social factors on CI-OPAT outcomes remains unknown, and additional prospective studies are needed to compare outcomes of CI-OPAT versus conventional OPAT.

This study has a few limitations. First, it is a single-center retrospective study. While our data demonstrate favorable safety outcomes, careful patient selection for CI-OPAT is required, and prospective studies will be beneficial to identify optimal inclusion and exclusion criteria. Second, we are unable to provide the exact time required for CI-OPAT initiation for each patient in our study as interprofessional discussions regarding the logistics of treatment initiation may have happened in channels that are not accessible for retrospective review. From our clinical experience, we estimate that CI-OPAT starts typically occurred within 1–2 business days after an identified need for OPAT. Third, estimating the cost impact and potential savings was beyond the scope of the current study. The insurance status of CI-OPAT patients may affect clinical decisions regarding CI-OPAT if home infusion services or parenteral medications are cost prohibitive. CI-OPAT has the potential to contribute to evolving data regarding the value of ID clinics and OPAT programs [20–22]. In particular, CI-OPAT can help avoid unnecessary emergency department visits or hospital admissions, which can be associated with substantial cost savings to healthcare systems. Adequate resources for implementation of CI-OPAT programs are critical to the provision of safe and effective care in this model.

In conclusion, CI-OPAT requires interprofessional collaboration, including readily available vascular access specialists and pharmacy support. Initiation of OPAT from ID clinics for appropriately selected patients can avoid unnecessary emergency department visits and inpatient admissions.
